# Parameters of geochemical effect equation for lanthanides and their geochemical significance for a series of metamorphic coals

**DOI:** 10.1038/s41598-020-70477-1

**Published:** 2020-08-10

**Authors:** Jianye Yang, Shenjun Qin, Jing Li, Fei Xu

**Affiliations:** 1 College of Material Science and Engineering, Xi’an University of Science and Technology, Xi’an, 710054 China; 2 Key Laboratory for Resource Exploration Research of Hebei Province, Hebei University of Engineering, Handan, 056038 China; 3 College of Resources, China University of Geosciences, Wuhan, 430074 China

**Keywords:** Geochemistry, Geology

## Abstract

The "geochemical effect of lanthanides" is a new concept proposed by the authors during the past decade. This concept reflects lanthanide shrinkage in elemental geochemistry, and it is statistically quantifiable. However, the geological significance of the various parameters of the equations obtained following quantization is not entirely clear. Cooperation and discussions from scholars in related research fields of rare earth elements in geology are required. In the present paper, from the perspective of coal geochemistry, the metamorphic coal seam of C2 series in Fengfeng Mine of Handan Coalfield in Hebei Province, China was used as an example. The geochemical significance of parameters in geochemical effect regression equation for lanthanides was evaluated, and two new formulas (regression equations) that characterized the geochemical behaviors of lanthanides were proposed. On this basis, concepts related to the geochemical effect of lanthanides, such as “individual parameters”, “parameters in common”, “two-sided parameters”, and the “deviation value” of lanthanides, were proposed. In this study, it was proved that the goodness of fit for all types of function regression equations for lanthanides and the radii of their trivalent ions, and the “deviation value” of lanthanides, were all “individual parameters” that could indicate the post-modified geological environment of C2 coal seam, such as the influence from magmatic-hydrothermal fluids. A covariant figure was constructed according to these individual parameters and other indexes, and the C2 coal seam in Handan was effectively divided into two different metamorphic series of A (C2 coal seam uninfluenced or slightly influenced by magmatic-hydrothermal fluids) and B (C2 coal seam strongly influenced and evidently changed by magmatic-hydrothermal fluids). Consequently, the scientific significance of all the parameters for lanthanides in an identifying series of metamorphic coals within the geochemical effect regression equation was further clarified.

## Introduction

The so-called “geochemical effect of lanthanides” phenomenon was established by the authors 10 years ago. This effect is a geochemical reflection of lanthanide contraction in the periodic table of elements, i.e., any quantifiable geochemical behaviors of lanthanides (for example: the normalized values of chondrite for lanthanides as well as the parameters characterizing the migration, distribution, and occurrence modes of lanthanides in geological bodies can all be considered as the results of quantifiable geochemical behaviors) usually have a strong linear relationship with certain atomic structural parameters (such as the radii of their trivalent ions)^1–8^. To investigate this relationship, lanthanides can be divided either into two parts (LREE and HREE), or take the 14 elements of lanthanides can be evaluated comprehensively. This relationship is described either by a linear regression function or through other functions, such as quadratic polynomial function, log function, exponential function, or power function. Usually, there are significant differences in the manifestations of the geochemical effect of lanthanides among geological bodies of different genetic types^5,6^. These differences likely result from the bodies’ unique geochemical states and processes, and so useful geological information may be acquired from them.

Within the currently available literature, there is no corresponding background information about this phenomenon. A few textbooks have mentioned that the complexation potential of the lanthanide elements enhances with an increase in their atomic numbers. However, these publications do not thoroughly probe into the atomic structural parameters of the lanthanide elements, such as the radii of their trivalent ions^9^.

Since the discovery of the geochemical effect of lanthanides, this research topic has received considerable attention from the authors, who have focused on the scientific significance and applications of this effect. In a recent article, we proposed a geochemical effect equation for lanthanides and explored the geochemical significance of certain parameters in all types of function regression equations^8^. However, being limited by the conditions at the time, we were unsuccessful in recognizing the geological significance of goodness of fit, which is a highly essential parameter within these regression equations. Concurrently, the explanation for the scientific significance of certain evaluated parameters was not sufficiently thorough. In this context, we took the late Paleozoic C2 coal seam of Handan Coalfield in Hebei Province of China as an ideal example and further evaluated the geological and geochemical significance of all parameters in the geochemical effect regression equation for lanthanides.

### Geological setting and previous research

**Figure 1:**
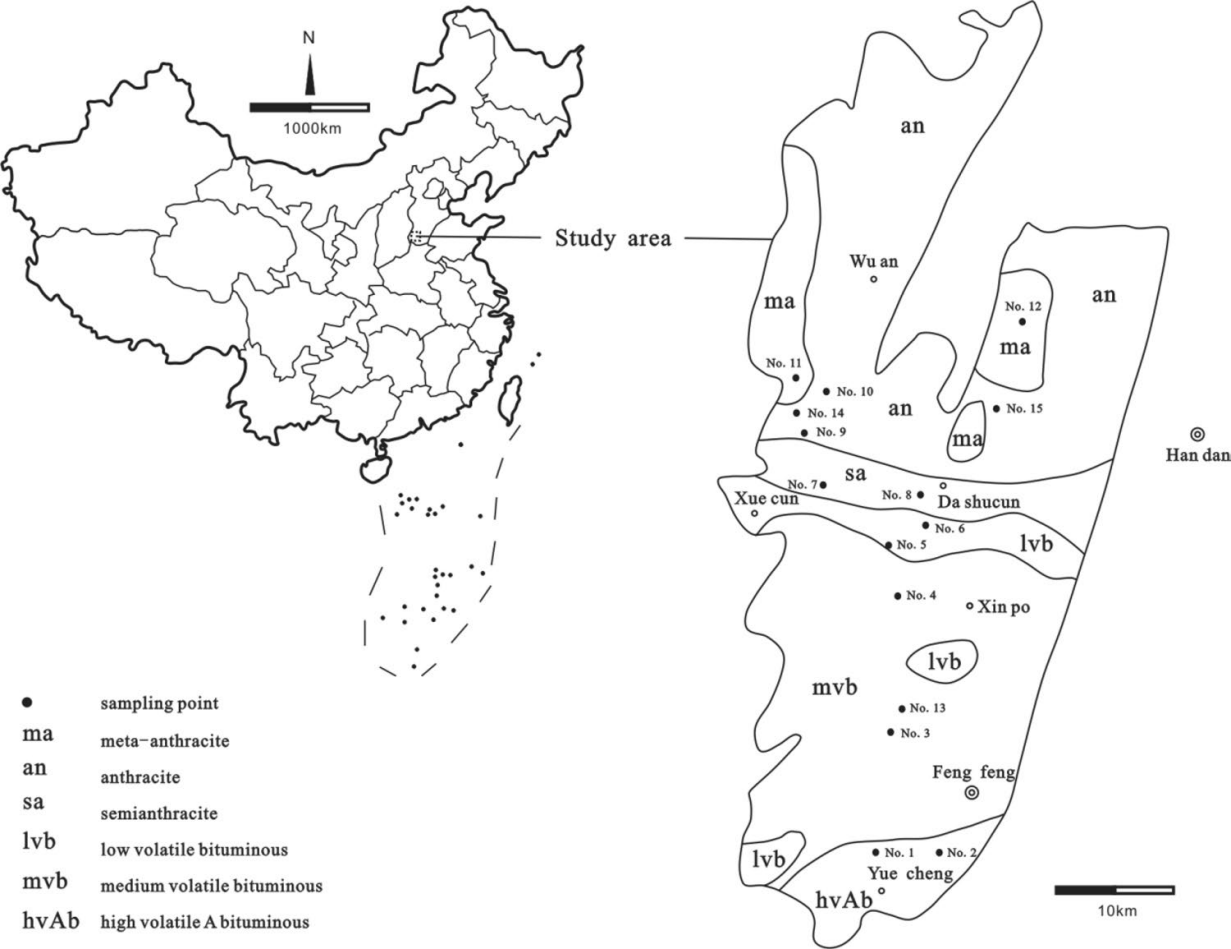
Location of the C2 coal seam and sampling location in the Fengfeng mine of the Handan Coalfield, Hebei Province (Dai and Ren, 2007^10^).

**Figure 2:**
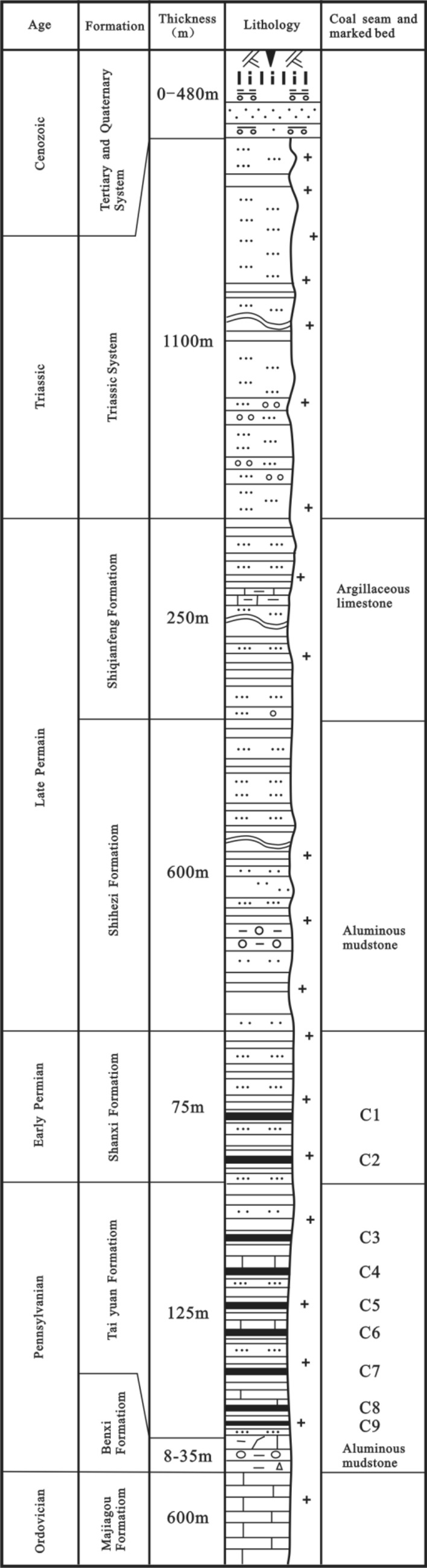
Profile of the sedimentary sequence of coal-bearing strata in the Fengfeng mine of the Handan Coalfield, Hebei Province (Dai and Ren, 2007^10^).

The Fengfeng Mine of Handan Coalfield (Hebei Province, China) was selected as the study area (Fig. 1), which contains a series of late Paleozoic coal-bearing strata (Fig. 2). Owing to the influence of Yanshanian magmatism on the C2 coal seam of the Shanxi Formation in the Early Permian, a series of thermal metamorphisms occurred almost continuously from south to north in the same coal seam; as a result, the coal gradually changed from low-rank bituminous to high-rank meta-anthracite (Fig. 1). To understand the influence of magmatism and coal's thermal metamorphism on the migration and distribution of elements in coal, systematic sampling and analyses have been conducted on coals with different ranks of metamorphism (*R*_max_ from 0.89% to 7.41%) in the C2 coal seam. The important parameters, including the concentration of lanthanides are shown in Table 1. Sampling and analysis methods were exhibited in the literature^10^. The previous work in this field has established a useful foundation for our study.

**Table 1:** Metamorphism, lanthanide abundance and related parameters of Late Paleozoic C2 coal seam in Fengfeng mine, China (Dai and Ren, 2007^10^).

Samples number	Coal seam	Coal rank	*R*_max_	La	Ce	Pr	Nd	Sm	Eu	Gd	Tb	Dy	Ho	Er	Tm	Yb	Lu	δCe	δEu	LREE	HREE	LREE/HREE	∑REE
No. 1	C2	hvAb	0.95	17.23	33.11	3.77	14.12	2.82	0.69	2.7	0.41	2.48	0.5	1.36	0.2	1.22	0.2	0.984	0.759	71.74	9.07	7.909	80.81
No. 2	C2	hvAb	0.89	17.2	33.08	3.74	14.09	2.79	0.64	2.67	0.38	2.45	0.47	1.33	0.17	1.19	0.18	0.988	0.712	71.54	8.84	8.092	80.38
No. 3	C2	mvb	0.98	12.98	23.63	2.59	9.43	1.71	0.38	1.63	0.26	1.52	0.31	0.86	0.14	0.83	0.15	0.977	0.691	50.72	5.7	8.898	56.42
No. 4	C2	mvb	1.39	15.52	30.6	3.64	13.9	2.94	0.62	2.86	0.47	2.75	0.55	1.6	0.24	1.55	0.25	0.976	0.649	67.22	10.27	6.545	77.49
No. 5	C2	lvb	1.58	14.55	26.65	2.79	9.99	1.75	0.38	1.68	0.26	1.51	0.31	0.88	0.14	0.82	0.14	1.002	0.673	56.11	5.74	9.775	61.85
No. 6	C2	lvb	1.79	17.89	32.35	3.42	12.19	2.13	0.54	2.04	0.31	1.8	0.38	1.12	0.17	1.08	0.2	0.991	0.786	68.52	7.1	9.650	75.62
No. 7	C2	sa	2.14	15.96	29.83	3.29	11.83	2.15	0.51	2.05	0.32	1.87	0.37	1.07	0.16	1.03	0.18	0.986	0.737	63.57	7.05	9.017	70.62
No. 8	C2	sa	2.25	24.37	43.54	4.36	14.66	2.59	0.55	2.56	0.39	2.27	0.45	1.3	0.19	1.18	0.2	1.012	0.648	90.07	8.54	10.54	98.61
No. 9	C2	an	4.51	13.58	26.88	3.04	11.88	2.35	0.5	2.45	0.4	2.28	0.48	1.42	0.21	1.39	0.24	1.002	0.632	58.23	8.87	6.564	67.1
No. 10	C2	an	6.15	20.68	34.37	3.29	11.04	1.83	0.46	1.95	0.28	1.61	0.33	0.93	0.14	0.82	0.15	0.998	0.739	71.67	6.21	11.54	77.88
No. 11	C2	ma	6.54	19.09	39.14	4.2	14.87	2.97	0.57	2.86	0.44	2.73	0.53	1.57	0.24	1.55	0.24	1.047	0.593	80.84	10.16	7.956	91
No. 12	C2	ma	7.41	13.88	26.81	2.84	10.51	1.74	0.36	1.74	0.25	1.42	0.3	0.84	0.13	0.8	0.15	1.023	0.628	56.14	5.63	9.971	61.77
Chondrite (Anders et al. 1989 Chondrite 10^–6^)				0.234	0.603	0.089	0.452	0.147	0.056	0.196	0.036	0.242	0.055	0.158	0.024	0.162	0.024						
Radius of lanthanides trivalent ion				1.061	1.03	1.01	1.03	0.96	0.95	0.94	0.92	0.91	0.89	0.88	0.87	0.86	0.85						

*hvAb* high-volatile A bituminous, *mvb* medium-volatile bituminous, *lvb* low-volatile bituminous, *sa* semianthracite, *an* anthracite, *ma* meta-anthracite.

### Research ideas and methods applied in this study

After numbering 12 coal samples according to the ranks of the C2 coal seam from low to high as No. 1–No. 12, their lanthanides were normalized with chondrite, and the results of normalization and the radii of their trivalent ions were subjected to a regression analysis with a linear function, quadratic polynomial function, log function, exponential function, and power function. The key obtained parameters are shown in Table 2. On this basis, the relationships between these parameters and other known geochemical parameters were utilized in investigating the geological and geochemical significance of these parameters.

**Table 2:** Important parameters obtained by regression of normalized lanthanide chondrite and the radius of their trivalent ion using various functions in the coals from Late Paleozoic C2 coal seam in Fengfeng mine, China.

Samples number	LREE lgf	HREE lgf	REE lgf	REE pgf1	REE egf	REE lgf	REE pgf2	LREE lrc	REE lrc1	REE prc1	REE erc	REE lrc2	REE liv	REE prc2	EivXv	pev
No. 1	0.823	0.888	0.788	0.926	0.928	0.769	0.917	477.4	264.3	1,982	0.0007	247.8	38.09	30.91	10.75	10.15
No. 2	0.827	0.888	0.791	0.927	0.932	0.772	0.921	482.6	267.0	1,984	0.0004	250.4	37.84	30.47	11.18	10.56
No. 3	0.814	0.653	0.74	0.916	0.876	0.718	0.860	375.8	192.0	1,684	0.0003	179.7	26.74	20.50	10.97	10.33
No. 4	0.839	0.786	0.785	0.926	0.883	0.765	0.869	427.1	231.5	1,768	0.0029	217.1	35.96	30.02	9.225	8.692
No. 5	0.805	0.749	0.731	0.909	0.881	0.709	0.866	428.9	216.9	1,925	0.0002	203.0	29.33	21.89	11.63	10.95
No. 6	0.798	0.454	0.726	0.911	0.871	0.704	0.854	518.0	263.4	2,390	0.0003	246.3	35.94	26.92	11.32	10.65
No. 7	0.814	0.690	0.752	0.917	0.892	0.731	0.878	460.7	241.1	2,034	0.0003	225.7	33.44	25.79	11.21	10.56
No. 8	0.778	0.802	0.708	0.892	0.880	0.687	0.865	722.3	357.9	3,281	0.0002	334.7	47.06	33.94	12.24	11.53
No. 9	0.844	0.558	0.760	0.925	0.847	0.739	0.830	384.2	198.3	1,660	0.0032	185.7	30.89	25.43	8.964	8.429
No. 10	0.755	0.736	0.683	0.882	0.886	0.662	0.871	606.4	293.7	2,844	0.00007	274.4	37.66	26.28	12.78	12.04
No. 11	0.810	0.774	0.749	0.906	0.860	0.729	0.845	560.9	292.2	2,400	0.0011	273.7	42.12	33.09	10.34	9.742
No. 12	0.831	0.579	0.749	0.920	0.876	0.727	0.860	418.7	214.7	1,847	0.0002	201	29.04	21.73	11.76	11.07

*lgf* linear goodness of fit, *pgf1* polynomial goodness of fit, *egf* exponential goodness of fit, *lgf* log goodness of fit, *pgf2* power goodness of fit, *lrc1* linear regression coefficient, *prc1* polynomial regression coefficient, *erc* exponential regression coefficient, *lrc2* log regression coefficient, *liv* log intercept values, *prc2* power regression coefficient, *EivXv* exponential independent variable X value, *pev* power exponent value.

## Results and discussion

### Correlation of parameters in the regression equation and parameters of REE

**Figure 3:**
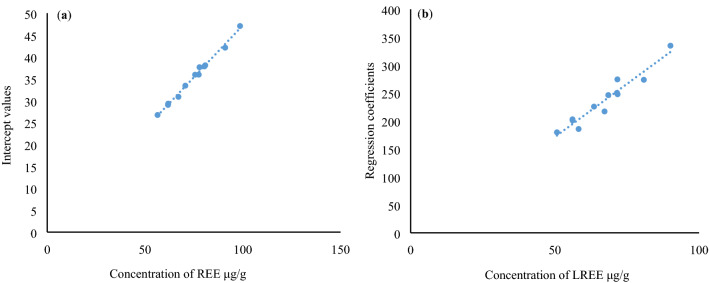
(**a**) Correlation between the intercept values obtained from log function regression for the geochemical effect of lanthanides in the C2 coal seam in Fengfeng Mine of Handan Coalfield in Hebei Province and the total concentration of REE, and (**b**) the correlation between the regression coefficients and concentration of LREE. The original data of REE refer to Table 1 (Dai Shifeng, 2007^10^), the regression coefficients refer to Table 2.

We took the relationship of lanthanides normalized with chondrite and the radii of their trivalent ions as an example and confirmed the previous findings. Specifically, the regression coefficients of each function (i.e., slope in the case of linear regression) from this study had significantly positive correlations with ∑REE and ∑LREE, and the power exponent of the power function has a significantly linear positive correlation with LREE/HREE^8^. In addition, this study also identified significant positive correlations between the intercept values obtained from log function regression and ∑REE, with a goodness of fit value of above 0.99 (Fig. 3a) and a value of 1 for some coal samples. The regression coefficients of the log function also showed significant positive correlations with the LREE concentration (with a goodness of fit of 0.9228, Fig. 3b). On this basis, a new regression equation was proposed:

1 $$F = k_{1} \sum LREE \, \ln \, r \, + \, k_{2} \sum REE,$$

where *F* is one of the geochemical behaviors of lanthanides, *r* is the radius of trivalent ion for lanthanides, and *k*_1_ and *k*_2_ are both proportionality coefficients. In the case of normalization of lanthanides with chondrite (the normalization process with chondrite was also taken as a geochemical process of the lanthanides, and more specifically their differentiation results relative to chondrite), the value of *k*_2_ can be approximated to 1.

In regression functions, few regression parameters have a correlation with ∑HREE. In this study, the regression coefficients of the power function for lanthanides in C2 coal seam of Handan and the radii of their trivalent ions had a significant positive correlation with ∑HREE (with a goodness of fit of 0.7033). Therefore, revising the equation as the following, produces a formula that is better than its counterpart in the literature^8^ (*F* = − *k*_1_∑REE *r*^*k*2 *f*^).

2 $$F \, = \, - K(k_{0}^{\prime } \sum LREE \, + \, k_{1}^{\prime } \sum HREE)r^{k2\; \, f} ,$$

where *r* represents the radii of trivalent ions of lanthanides, *f* is the differentiation degree of light (LREE) and heavy rare earth (HREE) elements, i.e., LREE/HREE, *k*_0_, *k*_1_, and *k*_2_ are all proportionality coefficients, and $k_{0}^{\prime }$ and $k_{1}^{\prime }$ are proportionality coefficients produced after extracting the common factor *K*.

### Three parameter types in the geochemical effect regression equation for lanthanides

Certain parameters listed in the above formulas, including regression coefficients, intercept values, and power exponents in the regression equation, are referred to as “parameters in common” in this paper, as the relationships of these parameters with ∑REE, ∑LREE, ∑HREE, and LREE/HREE are universal. Their geochemical significance conforms to the geochemical effect of lanthanides in coal and fits within geological bodies other than coal, such as the geochemical effect of lanthanides in magmatic rock (to be discussed later). Regardless of whether these samples were obtained from the same coal seam (or cognate magmatic rock, metamorphic rock or sedimentary rock, and so on) or different coal seams (or different sources and different kinds of magmatic rock, metamorphic rock, or sedimentary rock, and so on), the geochemical parameter characteristics of lanthanides (i.e., ∑REE, LREE/HREE, and so on) can always be described with the above parameters. Thus, the rule is “universally applicable”. This suggests that any geochemical behaviors or processes of lanthanides are limited by grand heat-tectonic events in geology and are strictly restrained by the internal microstructures of their ions in statistics. Moreover, these behaviors or processes are always closely related to the parameters of lanthanides, such as ∑REE, ∑LREE, ∑HREE, LREE/HREE, and so on. The geochemical behavior of every single lanthanide has a strict functional relationship with the radius of its trivalent ion, the total quantities of ∑REE and ∑LREE, and the differentiation degree of heavy and light rare earth elements. In other words, these are not purely individual behaviors. However, it is entirely possible that the values and ± of the proportionality coefficients in the above formulas (such as *K*, $k_{0}^{\prime }$, $k_{1}^{\prime }$, *k*_1_, *k*_2_, and so on) might vary with the different geochemical processes experienced by lanthanides, their different occurrence modes, their different geochemical environments, and so forth. Thus, they can be regarded as the “individual parameters” or “state parameters” in the above regression equations. For example, the complexation ability of lanthanides increases constantly with the reduction of the ionic radius. In this process, *k*_*1*_ in formula (1) should be negative. In contrast, in the fractional crystallization process of magma, the relationships of the LREE and HREE after normalization with chondrite and the radii of their trivalent ions usually presented a significant double-pitch-up pattern, i.e., the *K* values of the regression coefficients in these linear relationships should all be positive^6,9^. The latter research will show that the “deviation value” of lanthanides is also an “individual parameter”.

**Figure 4:**
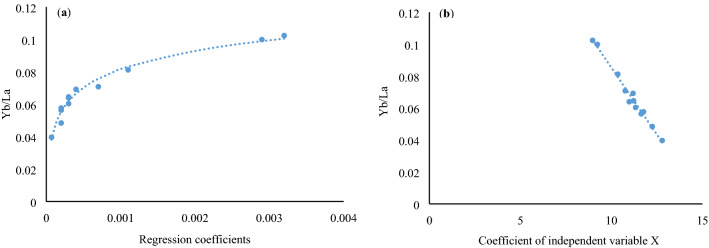
(**a**) Correlation between regression coefficients in the exponential function of geochemical effect for chondrite–lanthanides and Yb/La, and (**b**) the correlation between the coefficient of independent variable X (i.e., the coefficient of the radius of trivalent ion for lanthanides) and Yb/La in the C2 coal seam. The original data of REE refer to Table 1 (Dai Shifeng, 2007^10^), the regression coefficients refer to Table 2.

In this study, the linear regression coefficients in the relationship of the LREE after normalization with chondrite and the radius of its trivalent ion (hereinafter called “regression coefficients of geochemical effect equation for chondrite-linear lanthanides”) had significant or even highly significant positive correlations with concentration of Al, Si, La, Ce, and Pr (Table 3, with goodness of fit values of 0.4919, 0.5062, 0.9838, 0.8617, and 0.5559, respectively). Yb/La implies that the presence of multistage hydrothermal activity may also lead to the reallocation of trace elements, including rare earth element of coal-bearing rocks between coal seams and partings. Compared to partings, the high elemental ratios in coal seams (Yb/La, Nb/Ta, and Zr/Hf) are mainly caused by the re-precipitation of these elements (such as Yb, Nb, and Zr) in coal seams. In partings, these elements (Yb, Nb, and Zr) are more active in the leaching process and are easily leached, adsorbed by the underlying organic matter, and then precipitated in the underlying coal seam^11^. In this study, the regression coefficients in the exponential function of REE have a strong logarithmic relationship with Yb/La (Fig. 4a), whereas the coefficient of independent variable X in the exponential function shows a significant negative correlation with Yb/La (Fig. 4b). The power exponent obtained from the power function regression presents a similar relationship as shown in Fig. 4b. In addition, it indicates that the “parameters in common” such as certain functional regression coefficients, the coefficient of independent variable X (note that X refers to the radius of trivalent ions of lanthanides, which is regarded as a variable according to the regression formula) in the exponential function and power exponent of the geochemical effect of lanthanides, can also reflect geochemical processes under certain conditions; they also display certain characteristics of “state parameters”. This is because the ratios of certain elements inside lanthanides themselves have an environmental significance. Therefore, parameters such as the regression coefficients of exponential function for REE share the characteristics of both “parameters in common” and “individual parameters”, and these kinds of parameters can thus be called “two-sided parameters”.

**Table 3:** Ash yield (%, db), major and minor (wt %, db), and trace elements (μg/g, db) concentrations in the coals from the Fengfeng–Handan Coalfield (Dai and Ren, 2007^10^) and related ratios.

Element	No. 1	No. 2	No. 3	No. 4	No. 5	No. 6	No. 7	No. 8	No. 9	No. 10	No. 11	No. 12
Ash yield	18.5	16.2	19.5	16.1	18.6	20.6	22.4	19.7	23.4	26.6	30.3	22.1
Al_2_O_3_	6.49	5.24	7.21	5.53	6.85	6.86	7.21	12.57	6.93	8.22	5.48	5.47
SiO_2_	13.25	11.85	15.86	11.25	13.54	15.47	14.86	21.22	15.25	18.45	14.06	12.05
MgO	0.26	0.15	0.13	0.44	0.28	0.44	0.68	1.52	1.22	0.98	1.38	1.41
CaO	0.89	0.78	1.65	0.68	1.81	0.48	1.21	10.36	4.25	1.92	6.65	8.54
K_2_O	0.27	0.28	0.05	0.46	0.05	0.26	0.18	0.10	0.18	0.28	0.05	0.25
Na_2_O	0.09	0.11	0.43	0.19	0.16	0.08	0.09	0.07	0.10	0.05	0.23	0.35
TiO_2_	0.34	0.24	0.14	0.52	0.59	0.49	0.32	0.78	0.33	0.22	0.70	0.68
Fe_2_O_3_	0.60	0.65	0.78	0.54	0.72	0.89	0.84	1.25	1.84	2.65	8.56	1.48
MnO_2_	0.09	0.03	0.02	0.02	0.01	0.10	0.06	0.17	0.15	0.10	0.17	0.20
P_2_O_5_	0.03	0.03	0.03	0.41	0.02	0.02	0.51	0.83	0.16	0.22	0.76	0.49
Al	3.435	2.774	3.816	2.927	3.626	3.631	3.816	6.6545	3.668	4.3516	2.9011	2.8958
Si	6.188	5.534	7.407	5.254	6.323	7.224	6.94	9.91	7.122	8.616	6.566	5.627
Mg	0.157	0.09	0.078	0.265	0.169	0.265	0.41	0.917	0.736	0.591	0.832	0.85
Ca	0.636	0.558	1.18	0.486	1.294	0.343	0.865	7.407	3.039	1.373	4.755	6.106
K	0.224	0.232	0.042	0.382	0.042	0.216	0.149	0.083	0.149	0.232	0.042	0.208
Na	0.067	0.082	0.319	0.141	0.119	0.059	0.067	0.052	0.074	0.037	0.171	0.26
Ti	0.204	0.144	0.084	0.311	0.353	0.294	0.192	0.467	0.198	0.132	0.419	0.407
Mn	0.057	0.019	0.013	0.013	0.006	0.063	0.038	0.107	0.095	0.063	0.107	0.126
Fe	0.419	0.454	0.545	0.377	0.503	0.622	0.587	0.874	1.286	1.852	5.983	1.035
P	0.013	0.013	0.013	0.179	0.009	0.009	0.222	0.362	0.07	0.096	0.331	0.214
As	2.31	1.25	0.55	3.51	1.59	0.79	2.46	12.88	7.45	42.23	48.41	18.38
Ba	145.2	131.3	123.6	178.5	192.5	144.3	184.7	178.4	254.6	115.8	89.4	150.3
B	24.5	27.8	32.4	29.8	32.4	28.7	27.6	26.9	61.5	66.9	78.6	57.5
Be	2.11	1.67	1.22	2.34	2.55	2.57	1.74	0.65	1.80	0.88	1.52	2.20
Bi	0.29	0.18	0.13	0.43	1.04	0.24	0.49	0.99	0.17	0.45	0.20	0.41
Br	9.85	10.54	11.50	10.70	6.80	12.50	15.80	95.40	73.20	112.80	98.50	70.60
Ce	33.11	33.08	23.63	30.60	26.65	32.35	29.83	43.54	26.88	34.37	39.14	26.81
Cl	96.9	100.7	114.5	87.6	124.4	214.9	563.2	756.3	869.7	1,220.0	720.4	685.5
Co	2.80	2.45	2.30	1.50	1.00	3.50	2.80	4.20	14.60	29.20	33.70	18.90
Cr	18.40	10.55	19.10	9.60	15.80	18.70	21.50	10.50	14.70	13.30	19.10	15.40
Cs	0.12	0.22	0.85	0.14	0.91	0.54	0.25	0.11	0.09	0.04	0.18	0.09
Cu	8.54	11.58	26.50	15.40	9.87	14.60	14.70	11.20	65.40	121.50	181.40	90.80
Dy	2.48	2.45	1.52	2.75	1.51	1.80	1.87	2.27	2.28	1.61	2.73	1.42
Er	1.36	1.33	0.86	1.60	0.88	1.12	1.07	1.30	1.42	0.93	1.57	0.84
Eu	0.69	0.64	0.38	0.62	0.38	0.54	0.51	0.55	0.50	0.46	0.57	0.36
F	85.9	90.7	98.5	86.4	62.2	78.2	101.4	125.2	652.8	423.5	1,212.1	885.6
Ga	12.50	13.46	14.80	5.64	8.37	9.81	11.50	12.78	15.44	3.50	9.80	8.34
Gd	2.70	2.67	1.63	2.86	1.68	2.04	2.05	2.56	2.45	1.95	2.86	1.74
Hf	1.56	1.52	1.23	0.88	1.56	1.08	2.24	1.30	0.86	1.11	1.21	0.98
Hg	0.14	0.08	0.09	0.12	0.05	0.09	0.07	0.09	0.55	0.59	1.64	1.32
Ho	0.50	0.47	0.31	0.55	0.31	0.38	0.37	0.45	0.48	0.33	0.53	0.30
La	17.23	17.20	12.98	15.52	14.55	17.89	15.96	24.37	13.58	20.68	19.09	13.88
Lu	0.20	0.18	0.15	0.25	0.14	0.20	0.18	0.20	0.24	0.15	0.24	0.15
Mo	3.41	2.89	3.50	1.36	4.30	2.20	2.42	2.90	2.88	2.41	2.11	1.25
Nb	18.54	18.51	17.65	5.98	12.65	8.74	14.56	10.25	9.62	15.89	14.75	20.34
Nd	14.12	14.09	9.43	13.90	9.99	12.19	11.83	14.66	11.88	11.04	14.87	10.51
Ni	3.15	2.78	1.40	2.32	7.75	5.40	6.63	21.66	20.77	56.89	74.59	28.65
Pb	25.64	22.12	21.53	8.59	21.28	15.96	13.51	18.29	25.44	70.24	86.58	57.85
Pr	3.77	3.74	2.59	3.64	2.79	3.42	3.29	4.36	3.04	3.29	4.20	2.84
Rb	1.54	1.51	1.89	1.11	1.04	1.28	2.15	1.54	2.66	24.50	18.90	8.08
Sb	6.42	6.33	8.95	5.48	3.55	2.54	3.60	1.11	0.18	0.76	0.89	0.47
Sc	5.58	5.21	5.22	6.45	6.32	6.85	3.21	3.96	3.47	4.14	3.12	4.15
Se	3.58	3.44	2.12	1.25	2.56	2.76	1.91	1.93	1.05	0.94	11.85	0.68
Sm	2.82	2.79	1.71	2.94	1.75	2.13	2.15	2.59	2.35	1.83	2.97	1.74
Sn	7.85	8.95	11.23	8.45	10.68	12.04	8.37	11.21	6.34	8.67	13.09	6.52
Sr	187.5	100.5	199.3	168.0	288.4	197.1	236.1	220.5	589.5	1,024.5	1732.4	2,422.0
Ta	0.32	0.28	0.26	0.15	0.83	0.54	0.15	0.32	0.11	0.20	0.52	0.23
Tb	0.41	0.38	0.26	0.47	0.26	0.31	0.32	0.39	0.40	0.28	0.44	0.25
Th	9.70	10.20	11.66	3.65	10.25	18.40	3.42	10.07	3.10	8.66	6.70	6.71
Tm	0.20	0.17	0.14	0.24	0.14	0.17	0.16	0.19	0.21	0.14	0.24	0.13
U	1.54	1.25	0.75	1.21	1.25	2.38	1.85	8.50	25.51	32.48	24.32	21.59
V	55.56	54.88	93.65	52.87	65.82	54.09	20.75	28.54	18.49	16.58	15.44	22.36
W	1.57	1.04	1.94	0.89	1.15	2.22	0.59	1.76	2.40	2.36	2.41	2.54
Y	12.53	12.48	14.34	9.96	12.66	11.22	7.85	7.40	8.00	5.66	15.27	6.89
Yb	1.22	1.19	0.83	1.55	0.82	1.08	1.03	1.18	1.39	0.82	1.55	0.80
Zn	25.63	32.62	35.21	45.71	35.42	17.89	34.57	138.20	88.45	70.62	121.40	150.50
Zr	305.8	251.6	154.8	265.4	302.5	127.2	170.0	75.4	105.8	92.8	131.0	112.4
Th/U	6.298	8.16	15.54	3.016	8.2	7.731	1.848	1.1847	0.121	0.2666	0.2754	0.3107
Sr/Ba	1.291	0.765	1.612	0.941	1.498	1.365	1.278	12.359	2.315	8.8471	19.378	16.114
Th/Co	3.464	4.163	5.069	2.433	10.25	5.257	1.221	2.3976	0.212	0.2965	0.1988	0.3550
Zr/Hf	196.0	165.5	125.8	301.5	193.9	117.7	75.89	58	123.0	83.603	108.26	114.69
Zr/Nb	16.49	13.59	8.770	44.38	23.91	14.55	11.67	7.3560	10.99	5.8401	8.8813	5.5260
Yb/La	0.070	0.069	0.063	0.099	0.056	0.060	0.064	0.0484	0.102	0.0396	0.0811	0.0576
Nb/Ta	57.93	66.10	67.88	39.86	15.24	16.18	97.06	32.031	87.45	79.45	28.365	88.434
(SiO + Al_2_O_3_)/(Fe_2_O_3_ + CaO + MgO)	11.28	10.81	9.012	10.10	7.256	12.34	8.084	2.573	3.034	4.805	1.1779	1.5328

The goodness of fit in the above regression equation, which is also an “individual parameter”, likely depends upon the sources, geological origin, geological environment, geochemical process, occurrence mode, and other factors related to lanthanides. For example, the goodness of fit of a geochemical effect equation for chondrite-linear lanthanides of REE in the C2 coal seam shows a negative correlation with its LREE/HREE (with a goodness of fit of 0.7191). However, in other cases, this relationship may not hold true; one such instance was for the 616 basalt samples obtained from a sea bed^12^ (the detailed description is giving in another paper). This relationship cannot be established if lanthanides are normalized with a depleted mantle or enriched mantle, which represents a different geochemical process. However, in the case of No. 8 coal seam of Taiyuan Formation in Xishan of Taiyuan, Shanxi, China in the literature^13^, there is indeed such a relationship, but it is positive. Regardless of whether the 12 coal samples in C2 coal seam of Handan, or the five samples in No. 8 coal seam in Xishan of Taiyuan, Shanxi in the literature are used ^13^, all originate from the same coal seam of different metamorphic degrees. This likely implies that the linear goodness of fit is closely related to the differentiation degree of heavy and light rare earth elements only when the lanthanides are from same source and have undergone different evolutionary or metamorphic phases.

This study also found that the goodness of fit of the geochemical effect equation for chondrite-linear lanthanides of LREE also showed negative correlations with the concentrations of Al, Si, and La (with goodness of fit values of 0.4098, 0.5963, and 0.5328, respectively); that of REE presented similar correlations with the concentrations of Al, Si, and La (with goodness of fit values of 0.4084, 0.634, and 0.5328, respectively) and was positively correlated with the concentration of Zr (with a goodness of fit of 0.4147, which can reach 0.6858 after removing sample No. 5). The goodness of fit of the geochemical effect equation for chondrite-quadratic polynomial lanthanides of REE also showed negative correlations with the concentrations of Al, Si, and La (with goodness of fit values of 0.4296, 0.6369, and 0.5087, respectively), and exhibited a positive correlation with the concentration of U when the power function was used for fitting (with a goodness of fit of 0.6876). In general, in many cases, the goodness of fit of function regression usually shows a positive or negative correlation with several elements of lanthanides. This suggests that, in this example, some geochemical states (geochemical processes, occurrence modes, or origin evolution types) of lanthanides are related to the activity rules of some lanthanide elements as well as those of non-lanthanide elements especially Al and Si.

### “Deviation value” of lanthanides and its geochemical significance

Both previous studies and this present study indicate that, in some cases, geochemical behaviors of lanthanides in certain geological bodies should be more thoroughly described using a linear geochemical effect equation for lanthanides. In other cases, non-linear functions such as the log function, exponential function, power function, and quadratic polynomials are more appropriate (namely, the goodness of fit of the regression function is higher). At present, it is still difficult to offer a detailed and accurate explanation on this phenomenon. However, we believe that it might have been caused by different “geological or geochemical states”. In theory, the higher the goodness of fit for a functional relationship of the geochemical effect of lanthanides, the more singular the geochemical process, or “geological or geochemical states” such as source, origin, occurrence mode of the corresponding lanthanides. Certainly, in this study, it is only a scientific speculation or inference. Regarding the specific correspondence between the functional regression relationship and the geochemical state of lanthanides, studies and statistical analysis are still needed for further clarification. Based on the above speculation and inference and taking the geochemical effect equation for chondrite-linear lanthanides as an example, the investigation was conducted in two parts, i.e., LREE and HREE. The most ideal circumstance for the geochemical processes or occurrence modes characterized is a goodness of fit of 1. In general, this goodness of fit cannot be 1, because the geochemical behaviors or states of lanthanides, regardless of their distribution, migration process, or occurrence modes, are not purely singular. Therefore, the geochemical behavioral parameters of some lanthanides obtained from a regression formula with a goodness of fit 1 are only the “ideal value”. In most circumstances, the actual values of parameters for some geochemical behaviors of lanthanides do not match ideal values. This “deviation value” can be obtained from the difference between the parameter value obtained from the actual goodness of fit and the ideal value obtained by assuming the goodness of fit of 1. Table 4 shows the “deviation values” calculated from the geochemical effect equation for chondrite-linear lanthanides in 12 samples of the C2 coal seam in Handan. Tables 1 and 4 show that the deviation values of some elements for LREE are related to the anomaly of Ce. For example, the deviation value of Ce shows a negative correlation with δCe (goodness of fit = 0.6327), and positive correlations with ∑REE and ∑LREE (goodness of fit = 0.6571 and 0.6724, respectively). The same is true for the deviation value of Sm (the goodness of fit values for ∑REE and ∑LREE = 0.7915 and 0.737, respectively), which also has a positive correlation with ∑HREE (goodness of fit = 0.5898). The deviation value of Ho shows a negative correlation with ∑HREE (goodness of fit b = 0.44), while the deviation value of Yb exhibits a positive correlation with it (the goodness of fit being 0.4909). Thus, the deviation values of some elements for LREE can reflect their geological environments (such as the oxidation–reduction environments) and that the magnitudes of such deviation values are restrained overall by ∑REE, ∑LREE, or even ∑HREE. The deviation values of La and Eu also show positive correlations with LREE/HREE (goodness of fit = 0.614 and 0.8127, respectively), whereas those of Pr and Nd show negative correlations with LREE/HREE (goodness of fit = 0.7703 and 0.4152, respectively). This suggests that the differentiation degree of heavy and light rare earth is also one of the reasons causing the “deviation” of lanthanides.

**Table 4:** “Deviation value” of lanthanide elements and related parameters in the coals from Late Paleozoic C2 coal seam in Fengfeng mine, China (μg/g).

Samples number	La deviation value	Ce deviation value	Pr deviation value	Nd deviation value	Sm deviation value	Eu deviation value	Gd deviation value	Tb deviation value	Dy deviation value	Ho deviation value	Er deviation value	Tm deviation value	Yb deviation value	Lu deviation value	LREE Average deviation degree	HREE Average deviation degree	Deviation of (LREE-HREE)/2	Deviation of (La/Lu)
No. 1	9.09586	5.36058	2.32200	− 18.318	3.03063	0.95142	1.01156	− 0.1784	− 0.6304	− 0.6073	− 0.4169	− 0.0869	− 0.2194	1.12770	4.56909	0.2197	2.174660839	8.06583
No. 2	8.53278	5.05104	1.83870	− 18.644	2.96308	0.25157	1.14585	− 0.6290	− 0.3337	− 0.6376	− 0.0519	− 0.7284	0.23869	0.99185	4.52959	0.2342	2.14765836	8.60283
No. 3	7.81962	3.33980	0.75026	− 14.990	2.09754	1.01671	0.71078	0.12565	− 0.5023	− 0.6464	− 0.5381	0.10644	− 0.2993	1.03743	3.80163	0.2104	1.795578894	7.53742
No. 4	6.40838	4.25374	2.92107	− 15.750	3.41400	− 1.2290	0.93858	0.35569	− 0.7527	− 1.1747	− 0.4892	− 0.1327	− 0.0032	1.25476	3.69317	0.3036	1.694744708	5.10723
No. 5	9.04930	4.53313	0.24383	− 17.565	2.27386	1.45221	0.77040	0.01605	− 0.6106	− 0.6283	− 0.3516	0.20959	− 0.2151	0.81416	4.43582	0.1803	2.127756235	11.1148
No. 6	11.6133	5.07883	0.19323	− 21.606	2.19234	2.53585	1.26083	− 0.0351	− 0.8882	− 0.9298	− 0.4456	− 0.1990	− 0.3074	1.54705	5.53665	0.3096	2.613522082	7.50673
No. 7	9.00717	4.74101	1.42750	− 18.562	2.15510	1.25364	0.97360	0.10854	− 0.6285	− 0.9320	− 0.4795	− 0.2283	− 0.1280	1.31425	4.59494	0.2807	2.157080751	6.85343
No. 8	17.2419	7.98149	− 0.8196	− 31.795	3.97067	3.40842	1.17516	− 0.0007	− 0.8406	− 0.9986	− 0.3601	− 0.1393	− 0.1782	1.34145	8.17286	0.2925	3.9401751	12.8531
No. 9	5.76428	4.37603	1.61686	− 13.926	2.68392	− 0.5209	1.09847	0.32344	− 0.9677	− 1.0614	− 0.4243	− 0.3493	− 0.1393	1.51709	3.28177	0.3223	1.479728561	3.79956
No. 10	17.4178	5.08534	− 2.8398	− 27.490	2.99931	4.83778	1.17223	− 0.1873	− 0.8443	− 0.6972	− 0.3570	− 0.0018	− 0.3180	1.23138	7.48478	0.2594	3.61265925	14.1448
No. 11	8.180	9.1190	2.5886	− 22.89	3.6879	− 0.714	1.2321	− 0.2009	− 0.5772	− 1.3003	− 0.4558	0.0775	0.1951	1.0297	5.33635	0.3165	2.509893779	7.94425
No. 12	6.96	5.255	1.0572	− 15.9	1.946	0.733	1.2053	− 0.136	− 0.862	− 0.696	− 0.494	− 0.098	− 0.236	1.3235	3.82132	0.27323	1.77404595	5.26494

In general, the ratios of many elements can indicate sedimentary environments and geological origins in coal geology. For instance, a smaller ratio of Th/U in coal generally reflects a stronger hydrothermal influence on a coal seam^14^. High ratios of Yb/La, Nb/Ta, and Zr/Hf are mainly caused by the re-precipitation of Yb, Nb and Zr in the coal seam. In partings, these elements are active in the leaching process and can be easily leached, adsorbed by the underlying organic matter, and precipitated in the underlying coal seam^11^. The ratio of Nb/Ta can be used as the indicator to identify different evolutionary processes of magma^9,15^. The ratio of Sr/Ba increases with the distance away from the coast and can qualitatively reflect paleosalinity; specifically, the ratio of Sr/Ba < 1 in freshwater sediments, > 1 in marine sediments, and between 0.6 and 1 in blackish water facies^16^. The ash index (SiO_2_ + Al_2_O_3_)/(MgO + CaO + Fe_2_O_3_) mainly reflects the degree of reduction for a peat bog. When it is high, the reduction of media is weak in peat precipitation, and the aqueous nature of freshwater is evident; when it is low, the reduction of media is strong in peat precipitation^17^.

**Figure 5:**
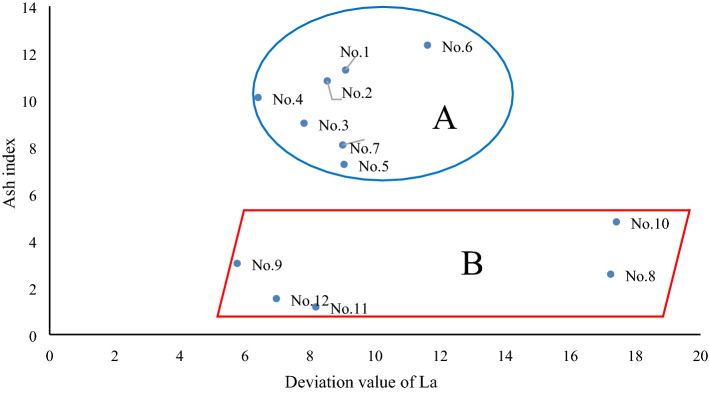
Covariant figure of deviation value of La–ash index in the C2 coal seam. The deviation value of La refers to Table 4, and the ash index refers to Table 3.

**Figure 6:**
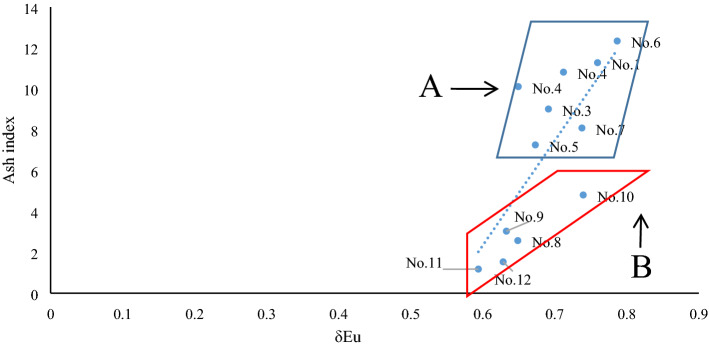
Covariant figure of δEu–ash index in the C2 coal seam. The value of δEu refers to Table 1, and the ash index refers to Table 3.

Based on the covariant figure of “deviation values” of lanthanides and some of the above elements' ratios, the 12 continuously metamorphous coal samples in the C2 coal seam of Handan can be divided into two different series. For instance, based on the covariant figure of the deviation value of the La-ash index in Fig. 5, these continuously metamorphous coal samples can be divided into series A (No. 1–No. 7), and series B (No. 8–No. 12). The literature^10^ indicates that samples No. 1–No. 7, from the coal seam are uninfluenced or slightly influenced by magmatic-hydrothermal fluids in C2, whereas samples No. 8–No. 12, from the coal seam are strongly influenced by magmatic-hydrothermal fluids in C2. The deviation values of La or Lu vs. the ash index can also constitute a similar covariant figure. Moreover, based on the covariant figure of the goodness of fit of chondrite-linear lanthanides and the ash index, these continuously metamorphous coal samples can also be divided into series A and series B. This suggests that the “deviation value” and goodness of fit are both useful “individual parameters” or “states parameters” that can indicate indicating their geochemical environments. Some “parameters in common”, such as the mentioned slope value (regression coefficient) of chondrite-linear lanthanides, power value after power function regression, and exponential value after exponent function regression, can all be used to distinguish the two series of A and B in the C2 coal seam in the covariant figure plotted from the “parameters in common” and ash index. Therefore, these “parameters in common” in fact fall within the scope of “two-sided parameters” described above. Other “parameters in common” observed by the predecessors, such as δEu, can also constitute a good covariant figure capable of identifying geological environments (Fig. 6). In Fig. 6, the samples of A series in the C2 coal seam, being uninfluenced or slightly influenced by magmatic-hydrothermal fluids are basically above the regression line, whereas those of B series in the C2 coal seam strongly influenced by magmatic-hydrothermal fluids are all below the regression line. Even the covariant figure constituted by ∑REE, ∑LREE, LREE/HREE, and the ash index can be used to distinguish A and B in the C2 coal seam. This result can also be inferred from the formulas (1) and (2).

**Figure 7:**
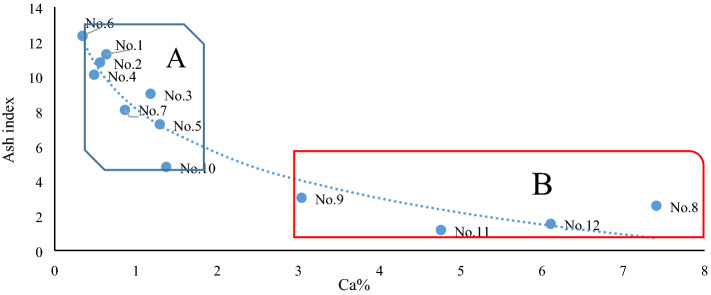
Covariant figure of Ca–ash index in the C2 coal seam. The concentration of Ca and the ash index refer to Table 3.

**Figure 8:**
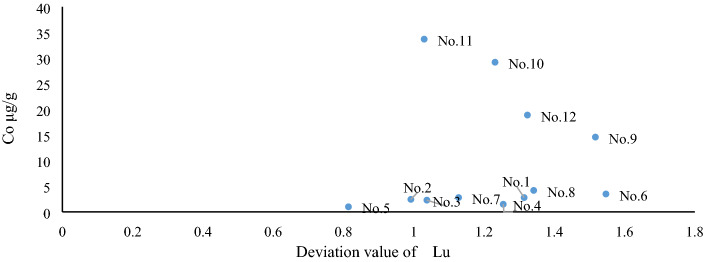
Covariant figure of the deviation value of Lu–Co in the C2 coal seam. The deviation value of Lu refers to Table 4, and the concentration of Co refers to Table 3.

The sixteen elements of B, F, Cl, Br, Hg, As, Co, Cu, Ni, Pb, Sr, Mg, Ca, Mn, Zn, and U in Table 3 and the ratios with certain geological or geochemical environmental significance (such as Sr/Ba, Th/U, and Th/Co), as well as other element ratios (such as Nb/Ta), can constitute a covariant figure capable of indicating a geological environment or origin through the “deviation values” of lanthanides or ash indexes. The above elements can be found within the hydrothermal fluid of magma^10^. For instance, the covariant figure of Ca%—ash index can reflect a strong logarithmic negative correlation between the indexes but also clearly distinguish between A and B of the C2 coal seam (Fig. 7). Certain covariant figures can also reflect slight differences in environmental influence. For example, although the covariant figure of the Lu deviation value-Co can clearly distinguish between A and B, it fails to classify sample No. 8, which is also strongly influenced by the hydrothermal fluids of magma, into Series B. According to a previous study (Figures 10 and 11, see reference)^10^, although some transition elements such as Co are found in the hydrothermal fluids of magma, in the phase of sample No. 8, the hydrothermalism is not strong enough. Herein, compared to the samples prior to No. 7, which are uninfluenced or slightly influenced by hydrothermal fluids of magma, there are few changes in elements, such as Co. Thus, sample No. 8 is not separated from Series A (Fig. 8).

### Environmental indication significance of parameter *R*_max_

**Figure 9:**
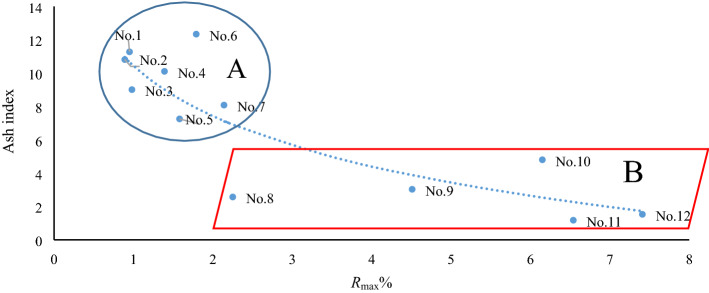
Covariant figure of *R*_max_–ash index in the C2 coal seam. The value of *R*_max_ refers to Table 1, and the ash index refers to Table 3.

*R*_max_, an important parameter used to characterize the metamorphic degrees of coal in the Handan C2 coal seam in Hebei, has no evident correlation with any parameter of the geochemical effect equation for lanthanides. However, the covariant figure constituted by *R*_max_ with other parameters still has certain environmental significance. For example, the covariant figure of the *R*_max_—ash index reflects the significant negative correlation between the *R*_max_ and ash index while clearly distinguishing between A and B for the C2 coal seam (Fig. 9). In turn, this completely indicates several super-metamorphic coal forms in the C2 coal seam with the superposition of the magmatic-hydrothermal process in Yanshanian based on plutonic metamorphism. For series A of samples No. 1–No. 7, the ash index reflects the changes in the reduction degree of peat bog upon the formation of C2 coal seam^17^. However, series B of samples No. 8–No. 12, it is more likely to reflect the result of magmatic-hydrothermal influence on the coal seam. Specifically, the large-scale injection of Ca and Mg in the hydrotherm causes the ash index of the coal seam to present an overall declining trend. The same applies to the covariant figures constituted by the above elements or elements' ratios related to magma hydrothermal fluid with the ash index or some parameters of the geochemical effect of lanthanides. Similarly, the covariant figure of *R*_max_-Th/U and *R*_max_-Th/Co can reflect the power function negative correlation between them and clearly distinguish between A and B for the C2 coal seam. Moreover, although *R*_max_ has no correlation with Sr/Ba, it can clearly distinguish between these two series as well.

## Conclusions

1. The geochemical significance of the parameters in the geochemical effect regression equation for lanthanides was systematically explained, and new descriptive formulas characterizing the geochemical behaviors of lanthanides were proposed. We speculate that different geochemical processes of lanthanides can be described with different regression functions; the higher the goodness of fit, the purer and more singular the different geochemical behaviors or processes of lanthanides will be.
2. Based on the above speculation, the concept of lanthanide deviation was proposed, and the parameters in the geochemical effect regression equation for lanthanides were roughly divided into three types: The first type is parameters acting in common, and its geochemical significance fits within any geochemical process experienced by any geological body other than coal. The second type is individual or state parameters, which are probably only applicable to certain geological bodies or geochemical environment that have stronger geochemical, environmental, and genetic significances. However, the difference between individual parameters and parameters in common is not “either this or that”. In this study, some parameters acted in common, such as the regression coefficients of some functions. Moreover, they had environmental indication significance under certain conditions. Therefore, the concept of “two-sided parameters” was established.
3. In this study, lanthanides had certain interactions with the elements from magmatic fluid or certain non-magmatic-hydrothermal-sourced elements such as Al and Si. Although the sources and changes of ∑REE and ∑LREE are not related with the magmatic-hydrothermal effect, they have certain interactions with the elements from the magmatic hydrothermal fluid. This manifests that the covariant figure with these elements can also be used to indicate different metamorphic series in the C2 coal seam. Moreover, this covariant figure conforms to the scientific logic inside regression formulas proposed in this study.
4. *R*_max_ in the C2 coal seam of Handan reflected the metamorphic degree of coal and indicated the influence of post-modification activities to coal through the combination with other indexes. For example, in the covariant figure of *R*_max_ and Sr/Ba or the ash index, the degree of the influence of later magmatic-hydrothermal fluid on the C2 coal seam was clearly reflected.

## Supplementary information

Supplementary Information.

## Data Availability

All the data including raw and processed data are available within the paper and Appendix.
